# Implication for Bone Marrow Derived Stem Cells in Hepatocyte Regeneration after Orthotopic Liver Transplantation

**DOI:** 10.1155/2013/310612

**Published:** 2013-09-10

**Authors:** N. Pilat, L. Unger, G. A. Berlakovich

**Affiliations:** ^1^Division of Transplantation, Department of Surgery, Medical University of Vienna, Waehringer Guertel 18-20, 1090 Vienna, Austria; ^2^Surgical Research Laboratories, Department of Surgery, Medical University of Vienna, Waehringer Guertel 18-20, 1090 Vienna, Austria

## Abstract

The liver has the outstanding ability to regenerate itself and restore parenchymal tissue after injury. The most common cell source in liver growth/regeneration is replication of preexisting hepatocytes although liver progenitor cells have been postulated to participate in liver regeneration in cases of massive injury. Bone marrow derived hematopoietic stem cells (BM-HSC) have the formal capacity to act as a source for hepatic regeneration under special circumstances; however, the impact of this process in liver tissue maintenance and regeneration remains controversial. Whether BM-HSC are involved in liver regeneration or not would be of particular interest as the cells have been suggested to be an alternative donor source for the treatment of liver failure. Data from murine models of liver disease show that BM-HSC can repopulate liver tissue and restore liver function; however, data obtained from human liver transplantation show only little evidence for liver regeneration by this mechanism. The cell source for liver regeneration seems to depend on the nature of regeneration process and the extent of injury; however, the precise mechanisms still need to be resolved. Current data suggest, that in human orthotopic liver transplantation, liver regeneration by BM-HSC is a rather rare event and therefore not of clinical relevance.

## 1. Introduction

Liver diseases are an important cause of morbidity and mortality in both Europe [1] and the USA [2], and the incidence for acute or chronic liver failure is rising due to hepatitis C infections, alcohol abuse, and hepatocellular carcinoma in cirrhosis [3]. Importantly, the liver represents the only vital organ (with exception of the brain) that cannot be replaced by a device because of the complexity of its functions. Orthotopic liver transplantation (OLT) is the treatment of choice for acute or chronic liver failure but is limited by general organ shortage, leading to increased mortality among patients on the waiting list. Although early graft loss as a consequence of primary nonfunction, hepatic artery thrombosis and acute rejection episodes is negligible due to constant improvement of surgical techniques, patient management, and immunosuppressive strategies, bacterial and fungal infections are a major problem being responsible for the majority of patient morbidity and graft loss in the first months after OLT. One-year survival rates for both the patient and graft survival are around 80% [4], with approximately 50% of all deaths happening within the first 6 months.

**Figure 1:**
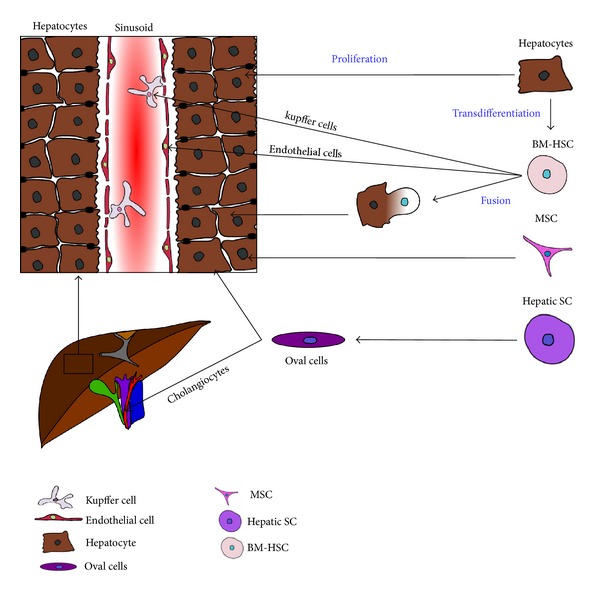
Cell sources for hepatocyte regeneration. Liver regeneration was suggested to result from proliferation, trans differentiation, and cell fusion, involving intrahepatic (hepatic SC, oval cells, and mature hepatocytes) and extrahepatic (BM-HSC, MSC) cell sources. BM-HSC: bone marrow derived hematopoietic stem cell, hepatic SC: hepatic stem cell, MSC: mesenchymal stem cell.

The concept of liver cell transplantation by hepatocyte infusion was suggested to be a promising alternative with significant advances over solid organ transplantation [5]. However, due to limited durability of functional benefit and limitations in acquisition and storage of mature hepatocytes, this approach is mainly used for inborn errors of metabolism in infants and as a bridging therapy to OLT [6]. Alternative cell sources have come into focus of research for cell based liver regenerative medicine in order to treat inherited or acquired liver diseases (Figure 1). Stem cell replacement strategies are therefore being investigated as an attractive alternative approach to liver repair. 

## 2. Bone Marrow Derived Hematopoietic Stem Cells as Source for Liver Regeneration

Bone marrow derived hematopoietic stem cells (BM-HSC) have the capacity to give rise to numerous cell populations, including hematopoietic cells, fibroblasts, endothelial cells, and mesenchymal stromal cells. However, the contribution of BM-HSC in hepatocyte regeneration remains unclear.

### 2.1. Data from Murine Models

Liver regenerative processes are mainly dependent on replication of already existing hepatocytes; however, it has been postulated that the source of hepatocytes depends on the nature of growth process and the extent of injury and may also involve bipotent precursor cells (oval cells) and BM-HSC. The so-called oval cells, which are able to participate in hepatocyte and cholangiocyte generation, have been suggested to be the progeny of BM-HSC as they share a panel of hematopoietic markers (c-kit, CD34, CD45, etc.) [7]. Conversion of BM-HSC to oval cells was observed in some animal models; however, hepatocytes derived thereof have been shown to be impaired in their repopulating capacity [8, 9].

Interestingly, BM-HSC have been demonstrated to repopulate liver and give rise to functional hepatocytes in a murine model of fatal hereditary tyrosinemia type I, which results in progressive liver failure. Fumarylacetoacetate hydrolase (FAH)-deficient mice were lethally irradiated and reconstituted with wildtype bone marrow (BM) in order to correct liver disease. FAH^−/−^ BM transplantation (BMT) recipients demonstrated substantial improvement compared to untreated control animals, and histological analysis confirmed the presence of donor-derived hepatocytes expressing FAH enzyme [10]. However, when the plasticity and *in vivo* cell fate specificity of BM-HSC were tested in lethally irradiated wildtype BMT recipients and parabiotic mouse models, chimerism was restricted to the hematopoietic system. Thus, production of nonhematopoietic cell types is not a typical function of normal BM-HSC, and transdifferentiation should be considered a very rare event [11]. More recent data demonstrated that the occurrence of donor-derived hepatocytes is not due to transdifferentiation but due to fusion of host hepatocytes with BM derived cells [12, 13]. BM-HSC derived hepatocytes were suggested to arise from cell fusion of donor HSC and recipient hepatocytes followed by reprogramming of HSC donor genomes, although the exact underlying mechanisms and the HSC type involved remain to be determined. Although cell fusion was shown to be capable of producing normally appearing hepatocytes and finally correcting the underlying metabolic disorder in the FAH^−/−^  model, the frequency of spontaneous fusion is very low, and the therapeutic potency for treatment of human liver diseases is questionable. 

Recently, it was shown that mobilization of host stem cells together with short course tacrolimus leads to operational tolerance and hepatic chimerism in a rat OLT model [26]. This experimental model of  “reverse chimerism” presents not only a potential tolerogenic approach for application in clinical OLT but also a potential therapy for hepatic regeneration. However, it was not determined whether chimerism was due to BM-HSC transdifferentiation or fusion, and mechanistic questions about tolerance induction remain open. 

### 2.2. Clinical Data from Sex-Mismatched Liver and BM Transplants

The state of microchimerism in solid organs after transplantation is well defined; however, with regard to the liver, the presence of donor-derived hepatocytes seems to be a rare event. Several groups have investigated the occurrence and fate of a recipient derived hepatocyte population after OLT. In most studies, sex-mismatched cases of OLT and BMT were used to assess origin of hepatic cells by visualizing X and Y chromosomes. Immunohistochemistry and fluorescent *in*-*situ* hybridization (FISH) were used to determine presence of BM-HSC derived hepatocytes. 

Whereas we and others have demonstrated that there is no or very little involvement of BM-HSC in liver regeneration after OLT [17, 19, 24], other studies reported findings of donor derived hepatocytes in patients that received liver or bone marrow transplants [14–16, 22, 23]. The frequency of BM-HSC derived hepatocytes varied between percentages <1% and 8% with some authors using sampling error corrections. Multiplication of frequency values with these correction factors lead to a reported frequency of BM-HSC derived hepatocytes of up to 43% [15]. Longitudinally performed biopsies suggest that hepatocyte chimerism is an early event (if it occurs at all) which is not correlated with the severity of injury [22, 23]. Several groups examined the status of hepatic cellular chimerism after OLT, reporting that substantial levels of chimerism are commonly found in macrophages, Kupffer cells and endothelial cells of the liver, and bile duct, whereas hepatocyte chimerism was seen only occasionally [18, 20, 21]. Interestingly, a more recent study reported hepatocyte chimerism to be present in high frequencies in pediatric liver allografts [25]. By using microdissection and microsatellite analyses, they revealed a higher percentage of chimerism in comparison to Y chromosome evaluation by FISH; however, hepatocyte chimerism was not correlated with injury or outcome and therefore, was not of clinical relevance (Table 1).

Results obtained from studies so far are controversial, which might be due to several reasons. (1) results usually were obtained in small groups of patients (4–24 patients). Due to the fact that biopsies were taken for clinical reasons (e.g., detection and scoring of rejection episodes or hepatitis C recurrence), timepoints of biopsies in relation to OLT and extent of injury are not comparable between patients. Prospective (multicenter) studies with a statistically relevant patient cohort and protocol biopsies would be needed to confirm findings and the biological significance of chimeric hepatocytes. (2) Different methods are used for the detection of chimerism. Some might claim that FISH methodologies lead to underestimation of chimerism because of sectioning and/or suboptimal hybridization efficiency. On the other hand, histological analysis without specific hepatocyte markers or without the use of confocal microscopy can lead to ambiguity errors as hybridization signals are commonly found in endothelial or Kupffer cell. Additionally, correction factors can introduce significant errors in false positive data. Generally, microchimerism detection on DNA level by using short tandem repeat (STR)/microsatellite analysis is suggested to be more specific; however, even using microdissection, it is very difficult to dissect hepatocytes only without including adjacent Kupffer cells or endothelial cells. (3) Different immunosuppressive regimens or other medications may influence BM-HSC mobilization and transdifferentiation. The difference between clinical studies and murine experimental models may also be due to the selection pressure. (4) The adult liver contains a significant number of hepatic stem cells and progenitor cells contributing to its enormous regenerative capacity [27]. Therefore, it is likely that circulating stem/progenitor cells or hepatocytes that are not removed at the time of OLT are responsible for hepatic chimerism.

It should also be noted that there is no evidence for significant repopulation of hepatic tissue with donor derived parenchymal cells in long-term allografts [28]. Considering the data available, it seems unlikely that BM-HSC transdifferentiation contributes significantly to physiological tissue regeneration or allograft acceptance after OLT.

## 3. Mesenchymal Stem Cells as a Source for Liver Regeneration

Mesenchymal stem cells (MSC) retain the potential to differentiate into functional hepatocyte-like cells and hepatic epithelial cells *in vitro* [29, 30] and, like BM-HSC, can be obtained in large quantities. Moreover, MSC have been shown to contribute to *de novo* generation of hepatocytes [31–33] and promote tissue regeneration by secretion of trophic molecules [32, 34, 35]. It has also been demonstrated that MSC are hypoimmunogenic and create an immunosuppressive microenvironment [36], thereby evading allogeneic rejection, a major problem in HSC transplantation [37]. Cotransplantation of MSC and HSC demonstrated synergistic effects of these 2 populations in bone vascularization [38] and heart failure [39]; moreover, MSC are suggested to promote HSC expansion and facilitate engraftment [40, 41]. Recently, MSC have been shown to be superior over BM-HSC in carbon tetrachloride induced liver injury regarding their homing abilities and modulation of chemically induced inflammation in the fibrotic liver. Surprisingly, the authors of this study reported no synergistic effects of MSC and HSC [42].

Despite the regenerative as well as immunomodulatory potential of MSC, the translation of experimental rodent studies into the clinical setting is hindered by safety concerns and the lack of molecular data regulating hepatocyte differentiation from MSC. Experimental difficulties arise from the fact that MSC can be obtained from various sources, including BM, umbilical cord blood, and adipose tissue, and, so far, unique molecular markers classifying MSC remain elusive. Due to the heterogeneous sources of MSC and different *in vitro* differentiation protocols, MCS cultures may contain different subpopulations with varying differentiation potential [43]. 

The *in vitro* differentiation potential of human MSC and functional capacity of hepatocytes derived thereof has been tested by *in vitro* functional assays demonstrating hepatocyte characteristics including albumin production, glycogen storage, urea secretion, uptake of low-density lipoprotein, and phenobarbital-inducible cytochrome P450 activity. Moreover, functionality has been demonstrated *in vivo* by successful engraftment in the liver followed by expression of HepPar1 and albumin [44, 45]. Importantly, they have also been shown to promote liver repair after hepatic damage *in vivo* [44, 46, 47].

MSC are suggested to have great potential in the treatment of liver diseases and may be even superior over primary hepatocytes due to their availability in large quantities; however, the underlying mechanisms of hepatocyte differentiation from MSC as well as the mechanisms driving hepatic engraftment still need to be resolved.

## 4. BM Derived Stem Cells and Fibrosis

Liver fibrosis, which is the main cause of many chronic liver diseases, is primarily characterized by an extensive deposition of extracellular matrix proteins (mainly type I collagen) in response to hepatic damage. The accumulation of collagen interferes with hepatic architecture and function which may subsequently progress into cirrhosis and liver failure [48]. BM derived cells have been proposed to contribute to collagen production and fibrosis in different models [49–51]; however, their contribution to scar tissue formation in the liver remains controversial [52–54]. 

With regard to regenerative medicine, BM-HSC and MSC have been proposed to be successful in supplying parenchymal cells; however, the fate of extracellular matrix remains largely unknown. Transplantation of BM cells was shown to exert antifibrotic effects and prevent formation of scar tissue in experimental rodent models by reducing carbon tetrachloride induced liver fibrosis [55, 56]. Bone marrow-derived cells were confirmed to produce antifibrotic collagenases including matrix metalloproteinase (MMP) 2, MMP 9, and MMP 13 and to simultaneously decrease expression of tissue inhibitors of MMPs (TIMPs) [55, 57].

Early phase clinical studies (using autologous bone marrow cells) for the treatment of fibrosis in advanced liver disease suggested the efficacy and safety of BMC therapy [58, 59]; however, larger randomized studies are required to find the optimal cell source and to evaluate therapeutic potency of this promising approach. A major problem hindering clinical translation of this promising approach is the lack of noninvasive techniques to quantify liver fibrosis in order to assess progression or reversal of disease.

## 5. Conclusions

Studies of hepatic regeneration processes gained prominence during the last decade, especially, since stem cell therapies are about to achieve a clinical impact. From a theoretical point of view, hepatocyte transplantation or transplantation of stem/progenitor cells that can restore liver function could represent an attractive alternative to OLT. Importantly, the source of hepatocytes always depends on the nature of growth process and the degree of injury. Whereas replication of preexisting hepatocytes is the common and most efficient way, blockade of this pathway can induce replication and differentiation of oval cells or, in rare cases, even the recruitment of BM-HSC. While the liver's exceptional capacity of self-renewal is known for centuries, the underlying mechanisms of liver repair still remain to be elucidated in order to develop therapeutic approaches based on cell therapy.

While transplantation of mature hepatocytes failed to achieve a clinical impact (mostly due to limited availability), BM-HSC and MSC seem to be promising candidates for regenerative cell therapy. Experimental studies from murine models and clinical pilot trials provided novel insights into different effects of transplantation of individual cell sources. We think that a more detailed understanding of the underlying mechanisms is a prerequisite for the development of cell therapies for liver disease. 

With respect to involvement of BM-HSC in liver transplantation, the studies discussed herein suggest that, although BM-HSC might be an important source for, for example, epithelial cells and Kupffer cells, differentiation to hepatocytes is a rather rare event. As “transdifferentiation”  has been proposed to occur by cell fusion instead [12], one would expect polyploidity among these cells, which was only reported in one study [14]. Moreover, genetic reprogramming following cell fusion was suggested to be involved in the generation of donor derived hepatocytes, challenging the paradigm of stem cell plasticity and transdifferentiation. So far, functional restoration of hepatocytes and subsequent cure of underlying disease could only be achieved in the murine FAH^−/−^  model, suggesting huge differences in liver repair between metabolic disorders due to genetic alterations and liver damage due to hepatitis or alcohol abuse. In this particular model, strong selective pressure model seems to favor cell fusion events resulting in hepatocyte-like cells that express an intact FAH allele; however, clinical relevance still needs to be confirmed. Cell fusion was also suggested to be involved in other famous examples of putative developmental plasticity [9, 14, 30, 60–63]; however, the field of stem cell plasticity is still up to debate [12].

With regard to therapeutics in order to replace lost or dysfunctional hepatocytes, we think that, although there is an exciting potential in the use of extrahepatic stem cells, there are many hurdles to overcome, and there is still a long way to clinical application. The therapeutic potential of stem cells is discussed for a variety of hepatic diseases, and at the moment there are several cell populations in the focus of research with all of them showing the ability to transform into hepatocytes in *in vitro* culture [64]; however, in terms of therapy, the most suitable cell population still has to be defined. One potential application causing much excitement is the use of stem cells for tissue engineering to seed the biologic components of artificial organs although this approach is far from clinical applicability. A more realistic approach for the next decade is the manipulation of stem cell signaling involved in the repair of liver tissue in order to allow self-regeneration of the damaged liver by genetic reprogramming of the therapeutic stem cell population.

Thus, although extrahepatic stem cells might offer promising resources for cell therapy, we think that physiological repopulation of the liver with BM-HSC derived hepatocytes after OLT is not of clinical relevance. Although the use of stem cells might be an attractive alternative to OLT, further studies are needed to examine their potential as liver repopulating cell source.

## Figures and Tables

**Table 1:** Selected clinical studies.

Reference	Pat. no.	Type of TX	D/R combination	Cases of hepatocyte chimerism	Hepatocyte chimerism %	Method	Additional findings
Alison et al. 2000 [14]	11 9	OLT HSCT	F→M M→F	Not specified	0.5–2%	FISH	Clonal growth of BM-HSC derived hepatocytes
Theise et al. 2000 [15]	4 2	OLT HSCT	F→M M→F	6/6	1–8% (4–43% adjusted)	FISH	Distribution of chimeric hepatocytes suggests different pathways of hepatic BM-HSC engraftment
Körbling et al. 2002 [16]	6	HSCT	M→ F	4/6	4–7%	FISH	Chimerism is not correlated with GVHD related tissue damage
Fogt et al. 2002 [17]	13	OLT	F↔M	none	0%	FISH	Hepatocyte chimerism is non frequent event in OLT
Kleeberger et al. 2002 [18]	9	OLT	Not specified	7/9	Not specified	STR	High frequency of cholangiocyte chimerism; hepatocyte chimerism associated with HCV recurrence
Wu et al. 2003 [19]	7 6	OLT Pediatric OLT	F→M	none	<0.4% putative hepatocytes	FISH	Recipient parenchymal cells are rare to nonexistent
ten Hove et al. 2003 [20]	5 11	OLT	F→M HLA I mismatch	1/5 Not specified	Not specified	FISH HLA-IH	Common endothelial and bile duct epithelial cell chimerism
Ng et al. 2003 [21]	10 5 2	OLT	F→M M→ F No sex mismatch	6/10 Not specified	<0.62% Not specified	FISH STR	High frequency of hepatic chimerism in Kupffer cells and macrophages
Idilman et al. 2004 [22]	11 5	OLT	F→M M→ F	6/5 5/5	0–2.4% 1.6–3.3%	FISH	BM-HSC derived hepatocytes are more common early after OLT, chimerism is not related to ACR
Idilman et al. 2007 [23]	9	OLT	F↔M	9/9	0.05–3.2%	FISH	BM-HSC derived hepatocyte repopulation is an early event
Pilat et al. 2012 [24]	14	OLT	F→M	none	0%	FISH	BM-HSC derived hepatocyte repopulation is not of clinical relevance
Aini et al. 2013 [25]	24	Pediatric OLT	Not specified	12/24	2.5–3.4%	FISH STR	Hepatocyte chimerism is suggested to be a common event and not correlated with hepatic injury

OLT: orthotopic liver transplantation, HSCT: hematopoietic stem cell transplantation, F: female, M: male, FISH: fluorescent in-situ hybridization, STR: short tandem repeat/microsatellite analysis, GVHD: graft-versus-host disease, HLA-IH: HLA specific immunohistochemistry, HCV: hepatitis C virus, and ACR: acute cellular rejection.
